# Association of hyperuricemia and a three-component lifestyle score with elevated cardiometabolic risk: a health-examination-based cross-sectional study

**DOI:** 10.3389/fendo.2026.1887627

**Published:** 2026-07-28

**Authors:** Kailin You, Qihui Chen, Lina Tang, Xiaoyang Zhang, Youqiong Xu

**Affiliations:** 1 The Affiliated Fuzhou Center for Disease Control and Prevention of Fujian Medical University, Fuzhou, China; 2 The School of Public Health, Fujian Medical University, Fuzhou, China; 3 Fuzhou Jin'an District Public Health Emergency Response Center, Fuzhou, China

**Keywords:** cross-sectional study, elevated cardiometabolic risk, hyperuricemia, interaction, three-component lifestyle score

## Abstract

**Background:**

Hyperuricemia (HUA) is increasingly recognized as being associated with cardiometabolic abnormalities, but its relationszhip with available lifestyle indicators in health-examination populations remains uncertain. This health-examination-based cross-sectional study examined whether hyperuricemia is associated with elevated cardiometabolic risk and whether the available three-component lifestyle score modifies this association.

**Methods:**

A health-examination-based cross-sectional study was conducted among 24,795 adult residents who attended the Xindian Town Health Center in Fuzhou, China. Participants underwent physical examinations, laboratory testing, and face-to-face questionnaire interviews. Hyperuricemia was defined as serum uric acid >420 umol/L in men and postmenopausal women or >360 umol/L in premenopausal women. Elevated cardiometabolic risk was defined as the presence of at least three of the following components: elevated blood pressure, abdominal obesity, abnormal glucose metabolism, elevated triglycerides, or reduced HDL-C. The TLS (0–3 points) comprised non-current smoking, non-excessive alcohol use, and adequate physical activity; dietary intake was not available and was therefore not included. Logistic regression and interaction analyses were adjusted for age and sex. Because the outcome was common, odds ratios were interpreted as odds rather than risk ratios.

**Results:**

The prevalence of non-current smoking, non-excessive alcohol use, and adequate physical activity was 90.9%, 95.6%, and 63.2%, respectively. The numbers of participants with TLS <=1, 2, and 3 were 1,322, 9,593, and 13,880, respectively. After adjustment for age and sex, a TLS of 2 or 3 was not significantly associated with overall elevated cardiometabolic risk compared with TLS <=1. Hyperuricemia was associated with higher odds of elevated cardiometabolic risk (OR = 1.66, 95%CI: 1.53-1.79). The multiplicative interaction between HUA and TLS was not statistically significant (OR = 0.96, 95%CI: 0.85-1.08, P = 0.462). No statistically significant additive interaction was detected (RERI = 0.15, 95%CI: -0.10 to 0.38; AP = 0.09, 95%CI: -0.06 to 0.22; SI = 1.30, 95%CI: 0.84 to 2.16).

**Conclusion:**

Hyperuricemia was associated with elevated cardiometabolic risk in this health-examination-based cross-sectional population. The available three-component lifestyle score did not show a statistically significant association with overall elevated cardiometabolic risk or robust interaction with HUA after age- and sex-adjustment. Because diet, socioeconomic factors, renal function, and specific urate-lowering therapy data were unavailable, these findings should be interpreted as hypothesis-generating associations rather than causal effects.

## 1 Introduction

Elevated cardiometabolic risk refers to the clustering of interrelated metabolic and cardiovascular risk factors, including obesity, elevated blood pressure, dyslipidemia, and abnormal glucose metabolism (1, 2). Metabolic syndrome (MetS) is a clinical construct that captures this clustering and is associated with higher future risk of cardiovascular disease (CVD) and type 2 diabetes (T2D) (3). Hyperuricemia has also received increasing attention because elevated serum uric acid is associated with cardiometabolic abnormalities and adverse cardiovascular outcomes (4). This condition arises from purine metabolism disorders and reduced uric acid excretion, and uric acid homeostasis depends partly on urate transporter regulation (5). With economic development and changing lifestyle patterns, the prevalence of hyperuricemia has increased in many populations, making it an important public health concern (1).

Currently, multiple large-scale prospective cohort studies provide compelling evidence linking hyperuricemia to cardiovascular and metabolic risks. Studies based on National Health and Nutrition Examination Survey (NHANES) data consistently demonstrate that elevated serum uric acid (SUA) levels correlate with increased cardiovascular and metabolic risk (6, 7). Furthermore, a multicenter retrospective cohort study has further revealed that individuals with hyperuricemia face a significantly elevated risk of adverse cardiovascular events (8). In the Chinese population, hyperuricemia has been identified as one of the key cardiometabolic risk factors (1), further supporting its potential association with cardiovascular risk.

Abnormal uric acid levels not only encompass hyperuricemia but also include hypouricemia. An observational study based on emergency department visits reveals that hypouricemia also exists among emergency patients, and it serves as a risk factor for cardiovascular diseases as well as cardiovascular and all-cause mortality. The effect of simple uric acid levels on these diseases exhibits a U-shaped pattern (9). Therefore, uric acid levels should be maintained at an appropriate level.

Faced with the growing burden of chronic diseases, prevention and management through lifestyle interventions have become a global consensus. In prior studies, healthy lifestyle scores often integrate multiple modifiable behavioral factors such as diet, physical activity, smoking status, alcohol consumption, weight control, and sleep duration (10, 11). The present dataset, however, contained only smoking, alcohol use, and physical activity variables suitable for scoring. Therefore, this study uses the term three-component lifestyle score (TLS) rather than implying a complete healthy lifestyle construct. The lack of dietary information is important because diet is a major determinant of both uric acid metabolism and cardiometabolic health.

Meanwhile, lifestyle influences uric acid metabolism with distinct specificity, with effects varying based on dietary patterns, exercise types, and environmental exposures (12). Within the Mediterranean dietary pattern, foods rich in flavonoids—such as citrus fruits and olive oil—significantly reduce serum uric acid levels (13); Resistance training stabilizes uric acid metabolism by improving insulin sensitivity, whereas intense exercise may induce transient uric acid elevation due to ATP depletion (14). Exposure to environmental toxins may suppress uric acid excretion by activating NLRP3 inflammasomes, exacerbating metabolic disorders and highlighting the importance of air pollution control in managing metabolic diseases (15).Furthermore, a healthy lifestyle influences cardiovascular metabolic risk through the aforementioned regulation of uric acid metabolism. These observations support evaluating lifestyle indicators together with HUA, while also requiring cautious interpretation when the lifestyle score is incomplete.

This study addressed three research questions. First, is HUA associated with elevated cardiometabolic risk in adults who underwent health examination at a town health center? Second, is the available TLS associated with elevated cardiometabolic risk and its components? Third, is there statistical evidence of multiplicative or additive interaction between HUA and TLS in relation to elevated cardiometabolic risk? We hypothesized that HUA is associated with elevated cardiometabolic risk. Analyses of TLS and interaction were considered exploratory because the available score did not include diet or other lifestyle domains.

## 2 Methods

### 2.1 Study subjects

This health-examination-based cross-sectional study involved permanent residents who attended health examinations at the Xindian Town Health Center in Fuzhou City, Fujian Province, between January 2017 and December 2024. The participants were not selected through random sampling of the entire community; therefore, the study population should be interpreted as residents participating in health examinations at a single town health center. The inclusion criteria were: (1) age >=18 years, (2) continuous residence in Xindian Town for >=6 months before the survey, (3) voluntary participation with signed informed consent, and (4) ability to independently complete questionnaires. Exclusion criteria included pregnancy, cognitive impairment, severe illness or disability preventing completion of the survey or examinations, and incomplete data on exposures, outcomes, or required covariates.

A total of 33,722 individuals were initially screened. After excluding individuals who did not meet eligibility criteria or had incomplete data required for the present analyses, 24,795 participants were included, yielding an effective response rate of 73.5%. The analytic dataset provided for this revision contained only complete cases, and row-level characteristics of the 8,927 excluded individuals were not available for comparison.

### 2.2 Data collection

Professionally trained investigators conducted face-to-face interviews using a self-developed questionnaire based on routine public health survey items. The questionnaire covered sociodemographic characteristics, medical history, behavioral and lifestyle factors, self-reported symptoms, and broad categories of medication use. Unified investigator training and standardized field procedures were used to reduce interviewer variation, but formal psychometric validation and reliability testing of the questionnaire were not conducted; this limitation is acknowledged below. Physical examinations and laboratory tests were performed to obtain measurements such as blood pressure, waist circumference, fasting blood glucose, serum uric acid, triglycerides (TG), and HDL-C. The study was approved by the Ethics Committee of Fuzhou Center for Disease Control and Prevention (approval number: 2022002). All participants provided written informed consent, and the procedures adhered to the Declaration of Helsinki.

### 2.3 Diagnostic criteria and related definitions

#### 2.3.1 Elevated cardiometabolic risk

According to the Guidelines for the Prevention of Cardiovascular and Metabolic Diseases, individuals meeting three or more of the following criteria were considered to have elevated cardiometabolic risk. This definition is adapted from commonly used metabolic syndrome criteria (NCEP ATP III and IDF) and is termed elevated cardiometabolic risk to reflect a risk-factor cluster in the examined population rather than a longitudinal disease endpoint. The components were: (1) elevated blood pressure (SBP >=130 mmHg and/or DBP >=80 mmHg and/or current antihypertensive medication use); (2) abdominal obesity (waist circumference >=90 cm in males and >=85 cm in females); (3) abnormal glucose metabolism (fasting blood glucose >=110 mg/dL [6.1 mmol/L], two-hour postglucose load blood glucose >=140 mg/dL [7.8 mmol/L], or current glucose-lowering medication use); (4) elevated triglycerides (fasting triglycerides >=150 mg/dL [1.7 mmol/L]); and (5) reduced HDL-C.

#### 2.3.2 Three-component lifestyle score

The TLS was constructed from the three available behavioral indicators: non-current smoking, non-excessive alcohol use, and adequate physical activity. Non-current smoking included never-smokers and former smokers. Non-excessive alcohol use was defined as no excessive alcohol consumption according to the questionnaire classification.

Adequate physical activity was defined as meeting the questionnaire category corresponding to regular activity consistent with the WHO recommendation of at least 150 minutes of moderate-intensity aerobic activity or at least 75 minutes of vigorous-intensity aerobic activity per week.

One point was assigned for each available favorable behavior, giving a score from 0 to 3. Scores of 0 and 1 were combined because of the small number of participants in these categories.

Because dietary intake, sleep, and other lifestyle domains were not collected, this score should be interpreted as a three-component indicator rather than a comprehensive healthy lifestyle score.

Accordingly, the terms TLS and available lifestyle score are used throughout the revised manuscript.

#### 2.3.3 Hyperuricemia

Hyperuricemia is a metabolic disorder involving elevated blood uric acid levels due to impaired purine metabolism or reduced uric acid excretion. In this study, HUA was defined as serum uric acid >420 umol/L in men and postmenopausal women or >360 umol/L in premenopausal women. Menopausal status was determined by self-reported cessation of menses for >=12 months.

### 2.4 Statistical analysis

All statistical analyses were performed using R software, version 4.3.2, and the revision-specific sensitivity analyses were replicated in Python for auditability. Continuous variables were expressed as the mean +/- standard deviation or the median (interquartile range), depending on distributional characteristics. Categorical variables were presented as counts (percentages), and group differences were assessed using chi-square tests. Logistic regression models were used to estimate odds ratios (ORs) and 95% confidence intervals (CIs). Because elevated cardiometabolic risk was common in this dataset, ORs were not interpreted as approximations of risk ratios.

To assess whether TLS modified the association between HUA and elevated cardiometabolic risk, we fitted models including HUA, TLS, and the HUA x TLS product term. Multiplicative interaction was evaluated using TLS as a continuous score. Additive interaction was evaluated by defining non-ideal TLS as <=2, with TLS = 3 as the reference, and estimating RERI, AP, and SI with 1000 bootstrap resamples. Primary models were adjusted for age and sex. BMI was not included in the final models because abdominal obesity was a component of the composite outcome, making BMI adjustment a potential overadjustment. Medication variables were not included in the primary models because the dataset contained broad self-reported medication categories without dose, duration, indication, or timing, and several medications may lie downstream of the outcome components. As a *post hoc* sensitivity analysis for possible reverse causality, we repeated selected age- and sex-adjusted models after excluding participants with self-reported hypertension, diabetes, cerebrovascular disease, heart disease, vascular disease, or coronary heart disease.

## 3 Results

### 3.1 Available lifestyle components, three-component lifestyle score, and elevated cardiometabolic risk among study participants

#### 3.1.1 Prevalence of three available lifestyle components among survey participants

Among the 24,795 participants, the prevalence of non-current smoking, non-excessive alcohol use, and adequate physical activity was 90.9% (22,535 individuals), 95.6% (23,712 individuals), and 63.2% (15,678 individuals), respectively. The numbers of participants with TLS <=1, 2, and 3 were 1,322 (5.3%), 9,593 (38.7%), and 13,880 (56.0%), respectively (**Table 1**).

**Table 1:** Distribution of the three available lifestyle components among survey respondents [n (%)].

Characteristics	Smoking status		Alcohol use		Physical activity	
	Non-current smoking	Current smoking	Non-excessive alcohol use	Excessive alcohol use	Insufficient physical activity	Adequate physical activity
Gender						
Male	7764 (34.5%)	2220 (98.2%)	8983 (37.9%)	1001 (92.4%)	3268 (35.8%)	6716 (42.8%)
Female	14771 (65.5%)	40 (1.8%)	14729 (62.1%)	82 (7.6%)	5849 (64.2%)	8962 (57.2%)
c2	3470.867		1278.814		116.880	
P value	<0.001		<0.001		<0.001	
Age						
18–	146 (0.6%)	23 (1%)	163 (0.7%)	6 (0.6%)	89 (1%)	80 (0.5%)
45–	2845 (12.6%)	312 (13.8%)	3047 (12.9%)	110 (10.2%)	1538 (16.9%)	1619 (10.3%)
60–	19544 (86.7%)	1925 (85.2%)	20502 (86.5%)	967 (89.3%)	7490 (82.2%)	13979 (89.2%)
c2	6.940		7.125		244.899	
P value	0.031		0.028		<0.001	
BMI (kg/m²)						
Underweight	313 (1.4%)	45 (2%)	343 (1.4%)	15 (1.4%)	132 (1.4%)	226 (1.4%)
Normal Weight	9178 (40.7%)	954 (42.2%)	9719 (41%)	413 (38.1%)	3704 (40.6%)	6428 (41%)
Overweight or Obese	13044 (57.9%)	1261 (55.8%)	13650 (57.6%)	655 (60.5%)	5281 (57.9%)	9024 (57.6%)
c2	7.817		3.615		0.332	
P value	0.020		0.164		0.847	
Occupational hazard exposure history						
No	22489 (99.8%)	2255 (99.8%)	23668 (99.8%)	1076 (99.4%)	9097 (99.8%)	15647 (99.8%)
Yes	46 (0.2%)	5 (0.2%)	44 (0.2%)	7 (0.6%)	20 (0.2%)	31 (0.2%)
c2	<0.001		8.586		0.047	
P value	1.000		0.003		0.828	

The table percentages describe the distribution of participant characteristics within TLS categories rather than the within-subgroup prevalence of TLS. Participants with TLS = 3 were more often female (63.9%) and aged >=60 years (89.1%). BMI distributions were similar across TLS categories, while occupational hazard exposure was rare in all TLS groups (**Table 2**).

**Table 2:** Three-component lifestyle scores of the survey respondents [n (%)].

Group	Three-component lifestyle score			chi2	P
	<=1	2	3		
Gender					
Male	1289 (97.5%)	3688 (38.4%)	5007 (36.1%)	1915.342	<0.001
Female	33 (2.5%)	5905 (61.6%)	8873 (63.9%)		
Age					
18-44	18 (1.4%)	79 (0.8%)	72 (0.5%)	174.254	<0.001
45-59	210 (15.9%)	1503 (15.7%)	1444 (10.4%)		
>=60	1094 (82.8%)	8011 (83.5%)	12364 (89.1%)		
BMI (kg/m2)					
Underweight	25 (1.9%)	138 (1.4%)	195 (1.4%)	2.708	0.608
Normal weight	542 (41.0%)	3889 (40.5%)	5701 (41.1%)		
Overweight or obesity	755 (57.1%)	5566 (58.0%)	7984 (57.5%)		
Occupational hazard exposure					
No	1316 (99.5%)	9577 (99.8%)	13851 (99.8%)	4.681	0.096
Yes	6 (0.5%)	16 (0.2%)	29 (0.2%)		

#### 3.1.2 Prevalence of three available lifestyle components among participants with elevated cardiometabolic risk

Among participants with elevated cardiometabolic risk, the proportions of non-current smoking, non-excessive alcohol use, and adequate physical activity were high, reflecting the older health-examination population and the cross-sectional nature of the data. These component-level patterns should not be interpreted as protective effects because participants with existing chronic conditions may have changed behaviors after diagnosis (**Table 3**).

**Table 3:** Distribution of available lifestyle components across elevated cardiometabolic risk and its components [n (%)].

Characteristics	Smoking status			Alcohol use			Physical activity		
	Non-current smoking	Current smoking		Non-excessive alcohol use	Excessive alcohol use		Insufficient physical activity	Adequate physical activity	
Elevated blood pressure									
No	1992 (8.8%)		194 (8.6%)	2120 (8.9%)		66 (6.1%)	875 (9.6%)		1311 (8.4%)
Yes	20543 (91.2%)		2066 (91.4%)	21592 (91.1%)		1017 (93.9%)	8242 (90.4%)		14367 (91.6%)
c2	0.137			10.087			10.792		
P value	0.712			0.001			0.001		
Abdominal obesity									
No	10528 (46.7%)		1140 (50.4%)	11145 (47%)		523 (48.3%)	4238 (46.5%)		7430 (47.4%)
Yes	12007 (53.3%)		1120 (49.6%)	12567 (53%)		560 (51.7%)	4879 (53.5%)		8248 (52.6%)
c2	11.285			0.641			1.866		
P value	<0.001			0.423			0.172		
Abnormal glucose metabolism									
No	9768 (43.3%)		970 (42.9%)	10306 (43.5%)		432 (39.9%)	4119 (45.2%)		6619 (42.2%)
Yes	12767 (56.7%)		1290 (57.1%)	13406 (56.5%)		651 (60.1%)	4998 (54.8%)		9059 (57.8%)
c2	0.135			5.244			20.465		
P value	0.714			0.022			<0.001		
TG elevated									
No	15515 (68.8%)		1442 (63.8%)	16239 (68.5%)		718 (66.3%)	6111 (67%)		10846 (69.2%)
Yes	7020 (31.2%)		818 (36.2%)	7473 (31.5%)		365 (33.7%)	3006 (33%)		4832 (30.8%)
c2	23.932			2.191			12.240		
P value	<0.001			0.139			<0.001		
Decreased HDL-C									
No	2344 (10.4%)		395 (17.5%)	2620 (11%)		119 (11%)	1023 (11.2%)		1716 (10.9%)
Yes	20191 (89.6%)		1865 (82.5%)	21092 (89%)		964 (89%)	8094 (88.8%)		13962 (89.1%)
c2	103.951			<0.001			0.418		
P value	<0.001			0.989			0.518		
Elevated cardiometabolic risk									
No	5309 (23.6%)		587 (26%)	5657 (23.9%)		239 (22.1%)	2237 (24.5%)		3659 (23.3%)
Yes	17226 (76.4%)		1673 (74%)	18055 (76.1%)		844 (77.9%)	6880 (75.5%)		12019 (76.7%)
c2	6.474			1.731			4.500		
P value	0.011			0.188			0.034		

For the composite elevated cardiometabolic risk outcome, the proportions with TLS <=1, 2, and 3 were 13.3%, 75.4%, and 76.9%, respectively. For several components, the direction of crude distributions varied across TLS categories, supporting the need for adjusted logistic models rather than descriptive interpretation alone (**Table 4**).

**Table 4:** Three-component lifestyle scores across elevated cardiometabolic risk and its components [n (%)].

Group	Three-component lifestyle score			chi2	P
	<=1	2	3		
Elevated blood pressure					
No	110 (8.3%)	903 (9.4%)	1173 (8.5%)	6.958	0.031
Yes	1212 (91.7%)	8690 (90.6%)	12707 (91.5%)		
Abdominal obesity					
No	654 (49.5%)	4487 (46.8%)	6527 (47.0%)	3.406	0.182
Yes	668 (50.5%)	5106 (53.2%)	7353 (53.0%)		
Abnormal glucose metabolism					
No	562 (42.5%)	4295 (44.8%)	5881 (42.4%)	13.689	0.001
Yes	760 (57.5%)	5298 (55.2%)	7999 (57.6%)		
Elevated triglycerides					
No	826 (62.5%)	6491 (67.7%)	9640 (69.5%)	30.938	<0.001
Yes	496 (37.5%)	3102 (32.3%)	4240 (30.5%)		
Reduced HDL-C					
No	204 (15.4%)	1104 (11.5%)	1431 (10.3%)	35.614	<0.001
Yes	1118 (84.6%)	8489 (88.5%)	12449 (89.7%)		
Elevated cardiometabolic risk					
No	328 (24.8%)	2359 (24.6%)	3209 (23.1%)	7.595	0.022
Yes	994 (75.2%)	7234 (75.4%)	10671 (76.9%)		

#### 3.1.3 Analysis of the association between three available lifestyle components and elevated cardiometabolic risk and its components

Age- and sex-adjusted logistic regression models were fitted for the three available lifestyle components. Adequate physical activity was positively associated with elevated blood pressure (OR = 1.28, 95%CI: 1.08-1.51) and elevated TG (OR = 1.12, 95%CI: 1.02-1.24) in the component-level models. Current smoking showed inverse associations with some components. These counterintuitive associations were treated as signals of possible reverse causality, selection bias, or residual confounding rather than as evidence of beneficial effects (**Table 5**).

**Table 5:** Association analysis of three available lifestyle components with elevated cardiometabolic risk and its components.

Group	Elevated blood pressure	Abdominal obesity	Abnormal glucose metabolism	Elevated triglycerides	Reduced HDL-C	Elevated cardiometabolic risk
	OR(95%CI)	OR(95%CI)	OR(95%CI)	OR(95%CI)	OR(95%CI)	OR(95%CI)
Model 1						
Smoking status						
Non-current smoking	1.00	1.00	1.00	1.00	1.00	1.00
Current smoking	1.03 (0.89-1.20)	0.86 (0.79-0.94)**	1.02 (0.93-1.11)	1.25 (1.15-1.37)**	0.55 (0.49-0.62)**	0.88 (0.80-0.97)*
Alcohol use						
Non-excessive alcohol use	1.00	1.00	1.00	1.00	1.00	1.00
Excessive alcohol use	1.51 (1.17-1.95)*	0.95 (0.84-1.07)	1.16 (1.02-1.31)*	1.10 (0.97-1.26)	1.01 (0.83-1.22)	1.11 (0.96-1.28)
Physical activity						
Insufficient physical activity	1.00	1.00	1.00	1.00	1.00	1.00
Adequate physical activity	1.16 (1.06-1.27)**	0.96 (0.92-1.02)	1.13 (1.07-1.19)**	0.91 (0.86-0.96)**	1.03 (0.95-1.12)	1.07 (1.01-1.13)*
Model 2						
Smoking status						
Non-current smoking	1.00	1.00	1.00	1.00	1.00	1.00
Current smoking	0.82 (0.69-0.98)*	1.00 (0.91-1.10)	0.88 (0.80-0.97)*	1.34 (1.21-1.48)**	0.82 (0.72-0.93)*	0.91 (0.82-1.02)
Alcohol use						
Non-excessive alcohol use	1.00	1.00	1.00	1.00	1.00	1.00
Excessive alcohol use	1.39 (1.06-1.81)*	1.09 (0.96-1.24)	1.08 (0.95-1.24)	1.06 (0.92-1.21)	1.66 (1.35-2.03)**	1.21 (1.04-1.42)*
Physical activity						
Insufficient physical activity	1.00	1.00	1.00	1.00	1.00	1.00
Adequate physical activity	1.08 (0.99-1.18)	0.96 (0.92-1.02)	1.10 (1.05-1.16)**	0.93 (0.88-0.98)*	1.09 (1.01-1.19)*	1.06 (0.99-1.12)

Model 1: Unadjusted; Model 2: Adjusted for age, sex, and mutual adjustment for the three available lifestyle components in the table; *P<0.05; **P<0.001.

In the *post hoc* sensitivity analysis excluding participants with known hypertension, diabetes, or cardiovascular/cerebrovascular disease, only 574 participants remained. In this restricted subset, adequate physical activity was no longer significantly associated with elevated blood pressure or reduced HDL-C, although the limited sample size reduced precision (**Supplementary Table 1B**). Among participants aged >=60 years, inverse associations between current smoking and some outcomes were attenuated for elevated blood pressure and abnormal glucose metabolism after the same exclusions, but the restricted sample remained small (**Supplementary Tables 1A, B**). These findings reinforce cautious interpretation of the cross-sectional component-level associations.

#### 3.1.4 Analysis of the association between three-component lifestyle scores and elevated cardiometabolic risk and its components

Using TLS <=1 as the reference category, age- and sex-adjusted models showed that TLS = 2 and TLS = 3 were inversely associated with elevated TG (OR = 0.75, 95%CI: 0.66-0.85; OR = 0.70, 95%CI: 0.62-0.79). TLS = 3 was positively associated with abnormal glucose metabolism (OR = 1.14, 95%CI: 1.01-1.28). Neither TLS = 2 nor TLS = 3 was significantly associated with overall elevated cardiometabolic risk after age- and sex-adjustment (**Table 6**).

**Table 6:** Association analysis of three-component lifestyle scores with elevated cardiometabolic risk and its components.

Group	<=1	2		3	
	OR(95%CI)	OR(95%CI)	P	OR(95%CI)	P
Model 1					
Elevated blood pressure	1.00	0.87 (0.71-1.07)	0.200	0.98 (0.80-1.21)	0.871
Abdominal obesity	1.00	1.11 (0.99-1.25)	0.066	1.10 (0.99-1.23)	0.089
Abnormal glucose metabolism	1.00	0.91 (0.81-1.02)	0.121	1.01 (0.90-1.13)	0.921
Elevated triglycerides	1.00	0.80 (0.71-0.90)	<0.001	0.73 (0.65-0.82)	<0.001
Reduced HDL-C	1.00	1.40 (1.19-1.65)	<0.001	1.59 (1.35-1.86)	<0.001
Elevated cardiometabolic risk	1.00	1.01 (0.89-1.16)	0.862	1.10 (0.96-1.25)	0.164
Model 2					
Elevated blood pressure	1.00	1.02 (0.82-1.26)	0.878	1.11 (0.89-1.37)	0.362
Abdominal obesity	1.00	0.95 (0.84-1.07)	0.386	0.92 (0.82-1.04)	0.171
Abnormal glucose metabolism	1.00	1.03 (0.91-1.16)	0.612	1.14 (1.01-1.28)	0.033
Elevated triglycerides	1.00	0.75 (0.66-0.85)	<0.001	0.70 (0.62-0.79)	<0.001
Reduced HDL-C	1.00	0.90 (0.76-1.06)	0.206	1.00 (0.85-1.18)	0.976
Elevated cardiometabolic risk	1.00	0.94 (0.82-1.08)	0.392	1.00 (0.88-1.15)	0.951

Model 1: Unadjusted; Model 2: Adjusted for age and sex.

### 3.2 Interaction between hyperuricemia and three-component lifestyle score on elevated cardiometabolic risk in study participants

#### 3.2.1 Association of three-component lifestyle score, hyperuricemia, and elevated cardiometabolic risk

In the model including TLS categories and HUA, HUA was associated with higher odds of elevated cardiometabolic risk after adjustment for age and sex (OR = 1.66, 95%CI: 1.53-1.79). In contrast, TLS = 2 (OR = 0.96, 95%CI: 0.83-1.10) and TLS = 3 (OR = 1.03, 95%CI: 0.89-1.18) were not significantly associated with overall elevated cardiometabolic risk compared with TLS <=1 (**Table 7**).

**Table 7:** Association of three-component lifestyle score and hyperuricemia with elevated cardiometabolic risk.

Group	Model 1		Model 2	
	OR(95%CI)	P	OR(95%CI)	P
Three-component lifestyle score				
<=1	1.00	–	1.00	–
2	1.03 (0.90-1.18)	0.674	0.96 (0.83-1.10)	0.517
3	1.12 (0.98-1.28)	0.085	1.03 (0.89-1.18)	0.713
Hyperuricemia				
No	1.00	–	1.00	–
Yes	1.67 (1.54-1.81)	<0.001	1.66 (1.53-1.79)	<0.001

Model 1: Unadjusted; Model 2: adjusted model included age and sex.

#### 3.2.2 Association between hyperuricemia and elevated cardiometabolic risk across three-component lifestyle score strata

Across TLS strata, HUA remained positively associated with elevated cardiometabolic risk, with adjusted ORs of 1.33 (95%CI: 0.98-1.79) for TLS <=1, 1.86 (95%CI: 1.63-2.11) for TLS = 2, and 1.56 (95%CI: 1.40-1.74) for TLS = 3. The estimate for TLS <=1 was less precise because this group was small (**Table 8**).

**Table 8:** Association of hyperuricemia with elevated cardiometabolic risk within three-component lifestyle score strata.

Group	Hyperuricemia				
	No	Yes			
		Model 1		Model 2	
		OR(95%CI)	P	OR(95%CI)	P
Three-component lifestyle score					
<=1	1.00	1.34 (0.99-1.81)	0.054	1.33 (0.98-1.79)	0.067
2	1.00	1.88 (1.66-2.14)	<0.001	1.86 (1.63-2.11)	<0.001
3	1.00	1.57 (1.41-1.75)	<0.001	1.56 (1.40-1.74)	<0.001

Model 1: Unadjusted; Model 2: Adjusted for age and sex.

#### 3.2.3 Multiplicative interaction between hyperuricemia and three-component lifestyle score on elevated cardiometabolic risk

When TLS was introduced as a continuous variable, the age- and sex-adjusted association between TLS and elevated cardiometabolic risk was not statistically significant (OR = 1.05, 95%CI: 0.99-1.10, P = 0.083). HUA remained positively associated with elevated cardiometabolic risk (OR = 1.85, 95%CI: 1.36-2.52, P<0.001). The HUA x TLS product term was not statistically significant (OR = 0.96, 95%CI: 0.85-1.08, P = 0.462), indicating no robust evidence of multiplicative interaction (**Table 9**).

**Table 9:** Multiplicative interaction analysis between hyperuricemia and three-component lifestyle score.

Variables	Model 1		Model 2	
	OR(95%CI)	P	OR(95%CI)	P
Three-component lifestyle score	1.08 (1.02-1.13)	0.005	1.05 (0.99-1.10)	0.083
Hyperuricemia	1.87 (1.37-2.54)	<0.001	1.85 (1.36-2.52)	<0.001
Hyperuricemia x lifestyle score	0.96 (0.85-1.08)	0.461	0.96 (0.85-1.08)	0.462

Model 1: Unadjusted; Model 2: Adjusted for age and sex.

#### 3.2.4 Additive interaction between hyperuricemia and non-ideal three-component lifestyle score on elevated cardiometabolic risk

Additive interaction analysis showed no statistically significant additive interaction between HUA and non-ideal TLS after adjustment for age and sex. The adjusted RERI was 0.15 (95%CI: -0.10 to 0.38), AP was 0.09 (95%CI: -0.06 to 0.22), and SI was 1.30 (95%CI: 0.84 to 2.16). Because all confidence intervals included the null values (RERI = 0, AP = 0, SI = 1), the data do not provide evidence that the joint association is greater than the sum of the individual associations. However, failure to detect additive interaction does not prove true additivity, especially given the width of the confidence intervals (**Table 10**).

**Table 10:** Additive interaction analysis of hyperuricemia and non-ideal three-component lifestyle score.

Model	RERI (95%CI)	AP (95%CI)	SI (95%CI)
Model 1	0.13 (-0.11 to 0.37)	0.08 (-0.07 to 0.22)	1.29 (0.83 to 2.15)
Model 2	0.15 (-0.11 to 0.38)	0.09 (-0.07 to 0.22)	1.30 (0.82 to 2.18)

Model 1: Unadjusted.

Model 2: Adjusted for age and sex. Non-ideal lifestyle was defined as TLS <=2, with TLS = 3 as reference. Bootstrap 95% CIs were based on 1000 resamples.

## 4 Discussion

This health-examination-based cross-sectional study found that HUA was consistently associated with elevated cardiometabolic risk among adults attending a town health center. After redefining the lifestyle measure as a three-component score based on available data, TLS did not show a statistically significant association with overall elevated cardiometabolic risk after adjustment for age and sex, and there was no statistically robust multiplicative or additive interaction between HUA and TLS. These findings support HUA as an important cardiometabolic marker in this population, while also showing that the incomplete lifestyle score should be interpreted cautiously.

A previous meta-analysis demonstrated that elevated SUA levels are significantly associated with all-cause mortality (relative risk [RR] 1.24; 95% CI 1.09–1.42) and cardiovascular mortality (RR 1.37; 95% CI 1.19–1.57) (16). Another study on emergency medicine has shown that elevated SUA is associated with an increased risk of ischemic stroke, suggesting that elevated uric acid can also affect the cerebrovascular system (17).Consistent with our findings, hyperuricemia is associated with elevated cardiometabolic risk in this population. This association can be explained through multiple biological mechanisms: studies indicate that elevated serum uric acid levels promote endothelial dysfunction, oxidative stress, and systemic inflammation, factors that collectively contribute to vascular injury (18, 19). These pathophysiological pathways provide a sound biological rationale for the elevated cardiometabolic risk observed in individuals with hyperuricemia.

The component-level lifestyle results were heterogeneous and included counterintuitive associations, particularly for physical activity and smoking. While higher levels of physical exercise were negatively correlated with elevated triglycerides and abdominal obesity, they were positively associated with increased blood pressure and reduced high-density lipoprotein cholesterol. This contradicts prior findings, such as a study indicating that individuals engaging in physical activity—regardless of intensity—exhibited positive changes in substances involved in blood pressure regulation, leading to reduced blood pressure (20). This apparent contradiction may stem from residual confounding factors such as age, baseline health status, or unmeasured comorbidities (21). For instance, in clinical practice, individuals with existing hypertension or metabolic syndrome are often advised to lower blood pressure through aerobic or resistance training (22). Such populations frequently appear as “high exercise + high blood pressure” in cross-sectional surveys because exercise serves as a therapeutic measure for their existing hypertension rather than a cause of elevated blood pressure. Meanwhile, Current smoking behavior was negatively associated with certain metabolic abnormalities, such as hypertension and reduced HDL cholesterol, contradicting previous studies (23, 24). This phenomenon may reflect “healthy smoker” bias or survival bias inherent in cross-sectional designs. Conversely, excessive alcohol consumption positively correlates with hypertension and reduced HDL cholesterol, consistent with existing evidence of alcohol’s dual effects on lipid metabolism and blood pressure regulation (25, 26).In the *post hoc* sensitivity analysis excluding known hypertension, diabetes, and cardiovascular/cerebrovascular disease, adequate physical activity was no longer significantly associated with elevated blood pressure or reduced HDL-C, but the restricted sample was small. These findings support treating the paradoxical associations as limitations of cross-sectional observational data rather than as substantive protective or harmful effects.

The interaction analyses also require conservative interpretation. The revised analyses did not show significant multiplicative interaction between HUA and TLS, and additive interaction indices had confidence intervals crossing the null. Therefore, the findings do not justify concluding that lifestyle statistically offsets or amplifies the HUA-cardiometabolic association in this dataset. At most, the results suggest that HUA remains associated with elevated cardiometabolic risk across TLS strata, and that studies with richer lifestyle measurement and longitudinal follow-up are needed to test effect modification more rigorously. In the present cross-sectional data, among individuals with hyperuricemia, those with TLS = 2 had the lowest observed prevalence of elevated cardiometabolic risk. Although some studies have shown that lifestyle interventions are still an adjustable approach to alleviate the metabolic consequences of hyperuricemia in situations where uric acid levels continue to rise (27), this may not be applicable to this study as the lack of dietary data in the lifestyle score limits the comprehensiveness of the assessment. Diet is widely recognized as a key determinant of uric acid levels and cardiometabolic health (28, 29) and its omission may affect the estimated association between lifestyle and cardiometabolic risk in this population.

Smoking and alcohol findings should be interpreted within the same framework. Current smoking showed inverse associations with some outcomes in older participants, but these results may reflect healthy smoker bias, survival bias, unmeasured disease severity, or behavior change after diagnosis. Excessive alcohol use and physical activity estimates may also be affected by residual confounding and measurement error. The present analyses therefore do not support clinical recommendations based on these component-level associations.

This study’s strengths include its large sample size, standardized health-examination measurements, and simultaneous evaluation of HUA, lifestyle indicators, and cardiometabolic risk components. The use of both multiplicative and additive interaction metrics provided complementary statistical perspectives. However, the study was cross-sectional and based on participants attending one town health center rather than a randomly sampled community cohort.

Several limitations should be emphasized. First, we could not adjust for dietary purine intake, socioeconomic status, renal function, specific urate-lowering therapy, medication dose or duration, or several comorbidities. Although broad medication categories were collected, they were not suitable for primary adjustment because indication and timing were unclear and some medications may be downstream of the cardiometabolic components. The absence of these variables may introduce residual confounding, and this should be taken into account when interpreting our findings. Moreover, we cannot ascertain whether any participants are undergoing uric acid-lowering therapy, thus making it impossible to evaluate potential biases. Second, the single measurement of serum uric acid limited our ability to assess the impact of uric acid fluctuations over time. Third, the TLS did not include dietary intake, sleep, socioeconomic status, or other important lifestyle domains; therefore, it should not be interpreted as a comprehensive healthy lifestyle score. Diet is the important modifiable determinant of uric acid metabolism and cardiovascular metabolic health. This may weaken the observed association and potentially introduce residual confounding factors. Fourth, the questionnaire was self-developed and did not undergo formal psychometric validation, which may introduce measurement error and social desirability bias. Fifth, the self-reported nature of lifestyle behaviors may introduce information bias and selection bias, thus affecting the outcomes. Sixth, row-level characteristics of excluded individuals were unavailable, so selection bias from incomplete data could not be evaluated. In addition, the cross-sectional design prevents temporal or causal inference, and reverse causality is particularly likely for physical activity and other behaviors that may change after diagnosis. Finally, some outcome definitions rely on single-visit measurements and medication information, which may produce misclassification.

## 5 Conclusion

This health-examination-based cross-sectional study found that HUA was associated with elevated cardiometabolic risk in adults attending a town health center. The available TLS, comprising non-current smoking, non-excessive alcohol use, and adequate physical activity, did not show a statistically significant association with overall elevated cardiometabolic risk after age- and sex-adjustment, and no statistically robust multiplicative or additive interaction with HUA was detected. Because diet, renal function, socioeconomic status, and specific urate-lowering therapy were unavailable, the findings should be interpreted as observational and hypothesis-generating. Future prospective studies with comprehensive lifestyle and medication data are needed to clarify temporal relationships and potential effect modification.

## Data Availability

The raw data supporting the conclusions of this article will be made available by the authors, without undue reservation.
