# Supplementary material for: Association of hyperuricemia and a three-component lifestyle score with elevated cardiometabolic risk: a health-examination-based cross-sectional study
Source: Front Endocrinol (Lausanne). 2026 Jul 28;17:1887627. doi: 10.3389/fendo.2026.1887627 (PMC13457293; doi:10.3389/fendo.2026.1887627)
Supplement: Supplementary file 1 [file Table1.docx]

**Supplementary Table S1. Sensitivity and age-stratified analyses for available lifestyle components.**

The analyses below use the revised terminology: current smoking, non-current smoking, adequate physical activity, and elevated cardiometabolic risk.

**Supplementary Table S1A. Age-stratified association analysis of current smoking and adequate physical activity with elevated cardiometabolic risk and its components.**

| Group | Elevated blood pressure | Abdominal obesity | Abnormal glucose metabolism | Elevated triglycerides | Reduced HDL-C | Elevated cardiometabolic risk |
| --- | --- | --- | --- | --- | --- | --- |
| Age 18-44 (n=169) | Age 18-44 (n=169) | Age 18-44 (n=169) | Age 18-44 (n=169) | Age 18-44 (n=169) | Age 18-44 (n=169) | Age 18-44 (n=169) |
| Model 1 | Model 1 | Model 1 | Model 1 | Model 1 | Model 1 | Model 1 |
| Smoking status | Smoking status | Smoking status | Smoking status | Smoking status | Smoking status | Smoking status |
| Non-current smoking | 1.00 | 1.00 | 1.00 | 1.00 | 1.00 | 1.00 |
| Current smoking | 2.90 (0.37-22.90) | 4.42 (1.65-11.89)* | 0.76 (0.31-1.82) | 5.28 (1.96-14.22)* | 0.15 (0.06-0.40)** | 1.66 (0.58-4.73) |
| Physical activity | Physical activity | Physical activity | Physical activity | Physical activity | Physical activity | Physical activity |
| Insufficient physical activity | 1.00 | 1.00 | 1.00 | 1.00 | 1.00 | 1.00 |
| Adequate physical activity | 0.89 (0.33-2.36) | 1.33 (0.72-2.45) | 0.51 (0.27-0.93)* | 0.49 (0.26-0.91)* | 1.13 (0.51-2.52) | 0.58 (0.30-1.12) |
| Model 2 | Model 2 | Model 2 | Model 2 | Model 2 | Model 2 | Model 2 |
| Smoking status | Smoking status | Smoking status | Smoking status | Smoking status | Smoking status | Smoking status |
| Non-current smoking | 1.00 | 1.00 | 1.00 | 1.00 | 1.00 | 1.00 |
| Current smoking | 1.60 (0.18-14.48) | 4.76 (1.64-13.88)* | 0.36 (0.13-0.97)* | 2.76 (0.94-8.09) | 0.28 (0.10-0.79)* | 0.86 (0.27-2.76) |
| Physical activity | Physical activity | Physical activity | Physical activity | Physical activity | Physical activity | Physical activity |
| Insufficient physical activity | 1.00 | 1.00 | 1.00 | 1.00 | 1.00 | 1.00 |
| Adequate physical activity | 0.90 (0.33-2.42) | 1.57 (0.83-2.97) | 0.42 (0.22-0.82)* | 0.48 (0.24-0.96)* | 0.89 (0.36-2.20) | 0.55 (0.27-1.09) |
| Age 45-59 (n=3157) | Age 45-59 (n=3157) | Age 45-59 (n=3157) | Age 45-59 (n=3157) | Age 45-59 (n=3157) | Age 45-59 (n=3157) | Age 45-59 (n=3157) |
| Model 1 | Model 1 | Model 1 | Model 1 | Model 1 | Model 1 | Model 1 |
| Smoking status | Smoking status | Smoking status | Smoking status | Smoking status | Smoking status | Smoking status |
| Non-current smoking | 1.00 | 1.00 | 1.00 | 1.00 | 1.00 | 1.00 |
| Current smoking | 1.27 (0.87-1.86) | 1.26 (1.00-1.60)* | 1.48 (1.17-1.88)* | 2.35 (1.85-2.97)** | 0.47 (0.34-0.64)** | 1.59 (1.19-2.12)* |
| Physical activity | Physical activity | Physical activity | Physical activity | Physical activity | Physical activity | Physical activity |
| Insufficient physical activity | 1.00 | 1.00 | 1.00 | 1.00 | 1.00 | 1.00 |
| Adequate physical activity | 0.95 (0.77-1.18) | 0.95 (0.83-1.10) | 1.20 (1.04-1.38)* | 0.83 (0.72-0.97)* | 1.07 (0.84-1.35) | 1.02 (0.88-1.20) |
| Model 2 | Model 2 | Model 2 | Model 2 | Model 2 | Model 2 | Model 2 |
| Smoking status | Smoking status | Smoking status | Smoking status | Smoking status | Smoking status | Smoking status |
| Non-current smoking | 1.00 | 1.00 | 1.00 | 1.00 | 1.00 | 1.00 |
| Current smoking | 1.39 (0.91-2.12) | 1.12 (0.86-1.46) | 0.95 (0.72-1.24) | 1.77 (1.36-2.32)** | 1.03 (0.73-1.47) | 1.24 (0.89-1.72) |
| Physical activity | Physical activity | Physical activity | Physical activity | Physical activity | Physical activity | Physical activity |
| Insufficient physical activity | 1.00 | 1.00 | 1.00 | 1.00 | 1.00 | 1.00 |
| Adequate physical activity | 0.92 (0.74-1.14) | 0.92 (0.80-1.06) | 1.17 (1.01-1.35)* | 0.82 (0.70-0.95)* | 1.13 (0.89-1.43) | 0.97 (0.83-1.14) |
| Age >=60 (n=21469) | Age >=60 (n=21469) | Age >=60 (n=21469) | Age >=60 (n=21469) | Age >=60 (n=21469) | Age >=60 (n=21469) | Age >=60 (n=21469) |
| Model 1 | Model 1 | Model 1 | Model 1 | Model 1 | Model 1 | Model 1 |
| Smoking status | Smoking status | Smoking status | Smoking status | Smoking status | Smoking status | Smoking status |
| Non-current smoking | 1.00 | 1.00 | 1.00 | 1.00 | 1.00 | 1.00 |
| Current smoking | 0.99 (0.83-1.17) | 0.80 (0.73-0.88)** | 0.96 (0.88-1.06) | 1.10 (1.00-1.22) | 0.57 (0.51-0.65)** | 0.80 (0.72-0.89)** |
| Physical activity | Physical activity | Physical activity | Physical activity | Physical activity | Physical activity | Physical activity |
| Insufficient physical activity | 1.00 | 1.00 | 1.00 | 1.00 | 1.00 | 1.00 |
| Adequate physical activity | 1.17 (1.06-1.29)* | 0.95 (0.89-1.00) | 1.11 (1.04-1.17)** | 0.93 (0.88-0.99)* | 1.03 (0.94-1.12) | 1.06 (0.99-1.13) |
| Model 2 | Model 2 | Model 2 | Model 2 | Model 2 | Model 2 | Model 2 |
| Smoking status | Smoking status | Smoking status | Smoking status | Smoking status | Smoking status | Smoking status |
| Non-current smoking | 1.00 | 1.00 | 1.00 | 1.00 | 1.00 | 1.00 |
| Current smoking | 0.79 (0.66-0.96)* | 0.96 (0.87-1.07) | 0.86 (0.78-0.96)* | 1.25 (1.12-1.39)** | 0.88 (0.77-1.01) | 0.88 (0.79-0.99)* |
| Physical activity | Physical activity | Physical activity | Physical activity | Physical activity | Physical activity | Physical activity |
| Insufficient physical activity | 1.00 | 1.00 | 1.00 | 1.00 | 1.00 | 1.00 |
| Adequate physical activity | 1.13 (1.02-1.25)* | 0.96 (0.91-1.02) | 1.09 (1.03-1.16)* | 0.95 (0.89-1.01) | 1.09 (0.99-1.19) | 1.07 (1.00-1.14)* |

Model 1: unadjusted. Model 2: adjusted for age, sex, current smoking, and adequate physical activity as applicable. *P<0.05; **P<0.001.

**Supplementary Table S1B. Post hoc sensitivity analysis after excluding participants with self-reported hypertension, diabetes, cerebrovascular disease, heart disease, vascular disease, or coronary heart disease.**

| Exposure | Elevated blood pressure | Abdominal obesity | Abnormal glucose metabolism | Elevated triglycerides | Reduced HDL-C | Elevated cardiometabolic risk |
| --- | --- | --- | --- | --- | --- | --- |
| Current smoking (n=574) | 0.66 (0.34-1.28) | 0.64 (0.34-1.19) | 0.87 (0.46-1.63) | 0.90 (0.46-1.78) | 0.83 (0.34-2.04) | 0.51 (0.28-0.94)* |
| Adequate physical activity (n=574) | 1.08 (0.73-1.61) | 1.29 (0.91-1.83) | 0.72 (0.50-1.03) | 1.06 (0.73-1.55) | 0.96 (0.51-1.82) | 1.16 (0.81-1.65) |

Models were adjusted for age and sex. The restricted sample was small; results are exploratory and should be interpreted cautiously. *P<0.05; **P<0.001.
